# US State Public Health Agencies' Use of Twitter From 2012 to 2022: Observational Study

**DOI:** 10.2196/59786

**Published:** 2025-01-03

**Authors:** Samuel R Mendez, Sebastian Munoz-Najar, Karen M Emmons, Kasisomayajula Viswanath

**Affiliations:** 1 Department of Social and Behavioral Sciences Harvard T.H. Chan School of Public Health Boston, MA United States; 2 Harvard Graduate School of Education Cambridge, MA United States; 3 Dana Farber Cancer Institute Boston, MA United States

**Keywords:** social media, health communication, Twitter, tweet, public health, state government, government agencies, information technology, data science, communication tool, COVID-19 pandemic, data collection, theoretical framework, message, interaction

## Abstract

**Background:**

Twitter (subsequently rebranded as X) is acknowledged by US health agencies, including the US Centers for Disease Control and Prevention (CDC), as an important public health communication tool. However, there is a lack of data describing its use by state health agencies over time. This knowledge is important amid a changing social media landscape in the wake of the COVID-19 pandemic.

**Objective:**

The study aimed to describe US state health agencies’ use of Twitter from 2012 through 2022. Furthermore, we organized our data collection and analysis around the theoretical framework of the networked public to contribute to the broader literature on health communication beyond a single platform.

**Methods:**

We used Twitter application programming interface data as indicators of state health agencies’ engagement with the 4 key qualities of communication in a networked public: scalability, persistence, replicability, and searchability. To assess scalability, we calculated tweet volume and audience engagement metrics per tweet. To assess persistence, we calculated the portion of tweets that were manual retweets or included an account mention. To assess replicability, we calculated the portion of tweets that were retweets or quote tweets. To assess searchability, we calculated the portion of tweets using at least 1 hashtag.

**Results:**

We observed a COVID-19 pandemic–era shift in state health agency engagement with scalability. The overall volume of tweets increased suddenly from less than 50,000 tweets in 2019 to over 94,000 in 2020, resulting in an average of 5.3 per day. Though mean tweets per day fell in 2021 and 2022, this COVID-19 pandemic–era low was still higher than the pre–COVID-19 pandemic peak. We also observed a more fragmented approach to searchability aligning with the start of the COVID-19 pandemic. More state-specific hashtags were among the top 10 during the COVID-19 pandemic, compared with more general hashtags related to disease outbreaks and natural disasters in years before. We did not observe such a clear COVID-19 pandemic–era shift in engagement with replicability. The portion of tweets mentioning a CDC account gradually rose and fell around a peak of 7.0% in 2018. Similarly, the rate of retweets of a CDC account rose and fell gradually around a peak of 5.4% in 2018. We did not observe a clear COVID-19 pandemic–era shift in persistence. The portion of tweets mentioning any account reached a maximum of 21% in 2013. It oscillated for much of the study period before dropping off in 2021 and reaching a minimum of 10% in 2022. Before 2018, the top 10 mentioned accounts included at least 2 non-CDC or corporate accounts. From 2018 onward, state agencies were much more prominent.

**Conclusions:**

Overall, we observed a more fragmented approach to state health agency communication on Twitter during the pandemic, prioritizing volume over searchability, formally replicating existing messages, and leaving traces of interactions with other accounts.

## Introduction

### Twitter as a Networked Public

This paper describes US state public health agencies’ activity on Twitter (subsequently rebranded as X) from 2012 through 2022. We used the theoretical framework of a networked public, an interactive space that networked technologies make possible, as well as the collection of people that inhabit it [1].

According to this theoretical framework, a networked public’s defining features are profiles, contact lists, and public communication tools. This framework outlines 4 emergent qualities of communication these features produce: scalability, persistence, replicability, and searchability. Twitter’s features were always open to users with potentially conflicting motivations. Viewed generously, this aligns with notions of Twitter’s democratization of health communication [2]. Viewed through a more critical lens, however, this illustrates how Twitter has been a site for both challenging and propagating inequities.

This tension was evident from Twitter’s 2006 launch, with dominant narratives of its Silicon Valley origins obscuring its relationship to open-source innovations from activists [3]. The hashtag illustrates such long-term tensions that exist in networked publics, between corporate practices and grassroots discourse, as well as between social networks with opposing goals. Hashtag use began as an informal organizational practice among power users before Twitter formally incorporated it as a feature in 2007 [4]. While activists eventually adopted hashtags for social justice campaigns like #MeToo and #BlackLivesMatter, and corporations adopted hashtags for advertising purposes, hashtags were also tools of political marginalization. For example, the use of the hashtag #ChineseVirus was a marker of anti-Chinese sentiment in March 2020, associated with anti-Asian hashtags more broadly after the US president’s use of the term “Chinese Virus” [5]. Hashtags can also be sites of conflict around public health topics, such as the coordinated flooding of the provaccine #DoctorsSpeakUp hashtag with antivaccine messaging in 2020 [6].

### Twitter as a State Public Health Tool

Though the use of Twitter in public health has been critiqued as “tweeting to the choir” [7], this type of communication toward professional audiences could reflect a viable platform-specific communication strategy. For example, the US Centers for Disease Control and Prevention (CDC) has recommended that public health communicators and organizational leaders use Twitter to reach reporters with crisis communication [8]. This could help explain why US health agencies’ COVID-19 tweets focused more on data than did their Facebook (Meta) posts [9].

Existing observational research reinforces this notion of Twitter as an information-focused public health communication tool. A study of state Medicaid program Twitter use between 2014 and 2019 found a similar informational focus, which garnered little audience engagement [10]. A study of Canadian public health agencies’ Twitter use during the first half of 2020 found a similar informational focus, even as more action-oriented tweets received higher audience engagement [11]. A study of health agency use of Twitter across 7 countries during the spring of 2020 found evidence of this trend internationally, with announcements and reporting being the most common tweet theme [12]. At a local level in the US, there have been experiments in using Twitter as a community service tool, such as in the case of the Chicago Department of Public Health’s foodborne illness response program [13].

Descriptive studies of US state public health agencies’ Twitter use, however, are either not comprehensive or are not up to date. Twitter was found to be an emerging platform for state public health agencies, based on a study covering a 2-month period in 2011 [14]. An early content analysis of state public health agency tweets in 2012 found a focus on the transmission of personal health advice [15] and a much higher Twitter adoption rate than local health departments around the same time [16]. A more recent study analyzed all state health agency tweets from a 4-month period around the emergency use authorization of the first COVID-19 vaccine in the United States [17]. Findings indicated a much higher volume of tweets than earlier studies, as well as a lack of key terms related to vaccination, inequities, and racism, lagging behind on-the-ground efforts to address COVID-19 racial health inequities.

We present an instrumental case study [18] to provide a more complete picture of state public health agency Twitter use long-term. We chose this approach as it aligns with our research interest in producing descriptive knowledge with theoretical implications beyond the individual case of Twitter. Our goal was to provide transferable knowledge to help identify trends and relationships between theoretical constructs in health communication. This knowledge is important amid a changing social media landscape in the wake of the COVID-19 pandemic. We follow STROBE (Strengthening the Reporting of Observational Studies in Epidemiology) guidelines [19] in reporting this study. The completed STROBE statement checklist is available in Multimedia Appendix 1.

## Methods

### Overview

We conducted a descriptive analysis of state public health agency tweets from 2012 through 2022, structured as an instrumental case study organized around an issue question [19]: How did US state public health agencies engage with Twitter as a networked public?

Our data came from a download of public health agency tweets using the “academictwitteR” R package to access the Twitter application programming interface (API) through the “Twitter API v2 for Academic Research” on February 3, 2023 [18]. We filtered the data to focus on tweets in English from state public health agencies. The language filter relied on labels available by Twitter’s proprietary API data. We identified tweets from the state-level public health agency in each state by leveraging associations between usernames and author IDs recorded during the data download process [20]. These accounts were initially identified through manual searches of state public health agency websites, as well as searches on Google and Twitter where necessary. Refer to Multimedia Appendix 2 for a list of account usernames and the associated state public health agencies. The study period begins on January 1, 2012, which allowed us to build on a previous study taking a random sampling approach to analyze state public health agency tweets in the same year [15]. The study period ends on December 31, 2022, which was the most recent full calendar year at the time of data collection. We did not account for missing data, as the API download method includes an exhaustive record of all published tweets from all specified accounts during the study period. We conducted this analysis using R (version 4.2.3, R Studio version 2023.06.1+524). The scripts enabling this analysis are available in an Open Science Foundation repository [21].

We calculated indicator variables to describe health agencies’ engagement with the key qualities of communication in a networked public: scalability, persistence, replicability, and searchability [1].

### Scalability

“Scalability” describes social media content’s hypothetical ability to reach an entire network. We assessed scalability by tweet volume and audience engagement. We calculated tweets per year, categorized into types: replies, retweets, or quote tweets (from their formal introduction in 2015) [22]. We further categorized replies into self-replies and other replies by comparing the author and in-reply-to user IDs. We used the regular expression “RT @” to identify “manual retweets,” a custom metric describing text copies of messages. Here, we pooled manual retweets with formal retweets. Finally, we calculated the mean, median, and IQR of states’ mean tweets per day each year, as well as the likes, replies, retweets, and quotes on non-retweets. “Non-retweets” include manual retweets, as they are unique posts rather than pointers to source tweets.

### Persistence

“Persistence” describes the digital records of interactions on networked publics. We assessed persistence by manual retweets. We also calculated mentions (a hyperlinked account username) among non-retweets, as their records do not depend on other accounts’ activity. We calculated the percentage of tweets that were manual retweets each year. We calculated the percentage of non-retweets with at least 1 mention each year.

### Replicability

“Replicability” refers to social media content’s infinite duplicability. We assessed replicability by retweets and quote tweets. We calculated the percentage of tweets that were formal retweets and quote tweets each year. We further calculated the percentage of tweets that were formal retweets of a CDC account, as well as the percentage of tweets including a CDC account mention. We used regular expressions to identify usernames starting with “CDC” or “NIOSH,” which comprised the majority of usernames listed as official CDC accounts [23]. For other accounts, we relied on exact matches with entire usernames.

### Searchability

“Searchability” refers to ways that social media content is findable, which we assessed by hashtag use. We calculated the percentage of tweets with at least 1 hashtag per year. We used regular expressions to extract hashtags and calculate the top 10 most common per year (after converting to lowercase). We further calculated the percentage of tweets per year using #flu or #hiv as hashtags with long-term national relevance.

### Ethical Considerations

The study data consisted of publicly available information from government institutional authors. We did not seek an ethics review and have no individual participant protections in place, as they are not relevant to this study design.

## Results

### Overview

We identified 570,335 tweets from state public health agency accounts in English from 2012 through 2022. All 50 states were represented in this data set. However, all 50 states were only active on Twitter in 2018 and 2019, as states gradually adopted Twitter during the study period, and 1 state published its last tweet in 2019. We included 52 accounts in this data set to account for 1 state creating a replacement public health agency account during the study period, as well as another state communicating through 2 accounts under its public health agency. An exhaustive summary data table is available in Multimedia Appendix 3.

### Scalability

**Figure 1:**
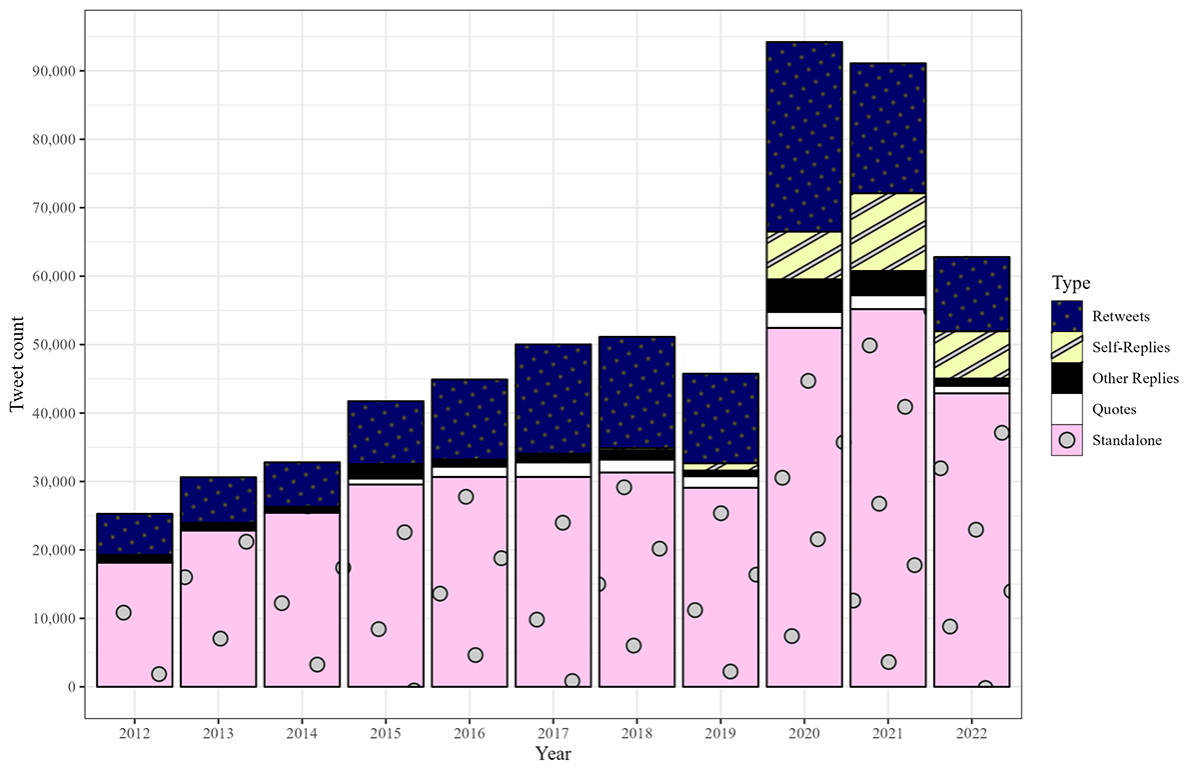
Stacked bar chart displaying an exhaustive enumeration of tweets from US state public health agency accounts for each year from 2012 through 2022, divided into 5 categories: retweets, self-replies, other-replies, quote tweets, and standalone tweets.

**Figure 2:**
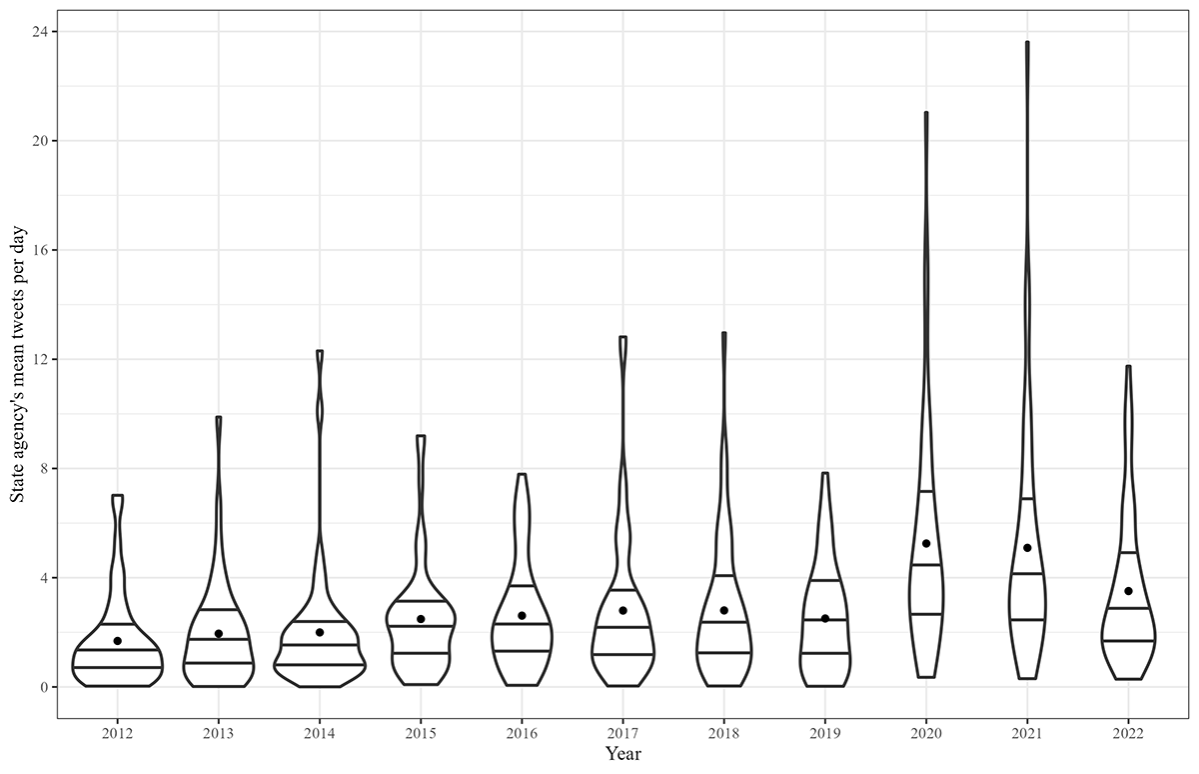
Among an exhaustive enumeration of tweets from US state public health agency accounts, violin plots displaying the distribution of the mean daily tweets per account, each year from 2012 through 2022. IQRs for each year are marked with horizontal lines. The mean of means for each year is marked with a dot.

In total, 41 states were active at any time in 2012, meaning they posted at least 1 tweet. This number increased each year, up to 50 in 2018. It decreased to 49 in 2020. The total number of tweets across all accounts increased from 25,276 in 2012 to 51,132 in 2018. The total rose to 94,205 in 2020 and decreased to 62,797 in 2022 (Figure 1). Overall, mean tweets per day rose from 1.7 in 2012 to 2.8 in 2018. It reached a maximum of 5.3 in 2020 and remained above the 2018 level. Figure 2 displays the distribution of mean tweets per day among active states each year. Delaware, North Carolina, Florida, Indiana, Pennsylvania, Massachusetts, and West Virginia each had the most tweets in a given year. Delaware, Florida, and West Virginia each had the most for multiple years in a row. Massachusetts was the most active in a single year, averaging >21 tweets per day in 2020.

**Figure 3:**
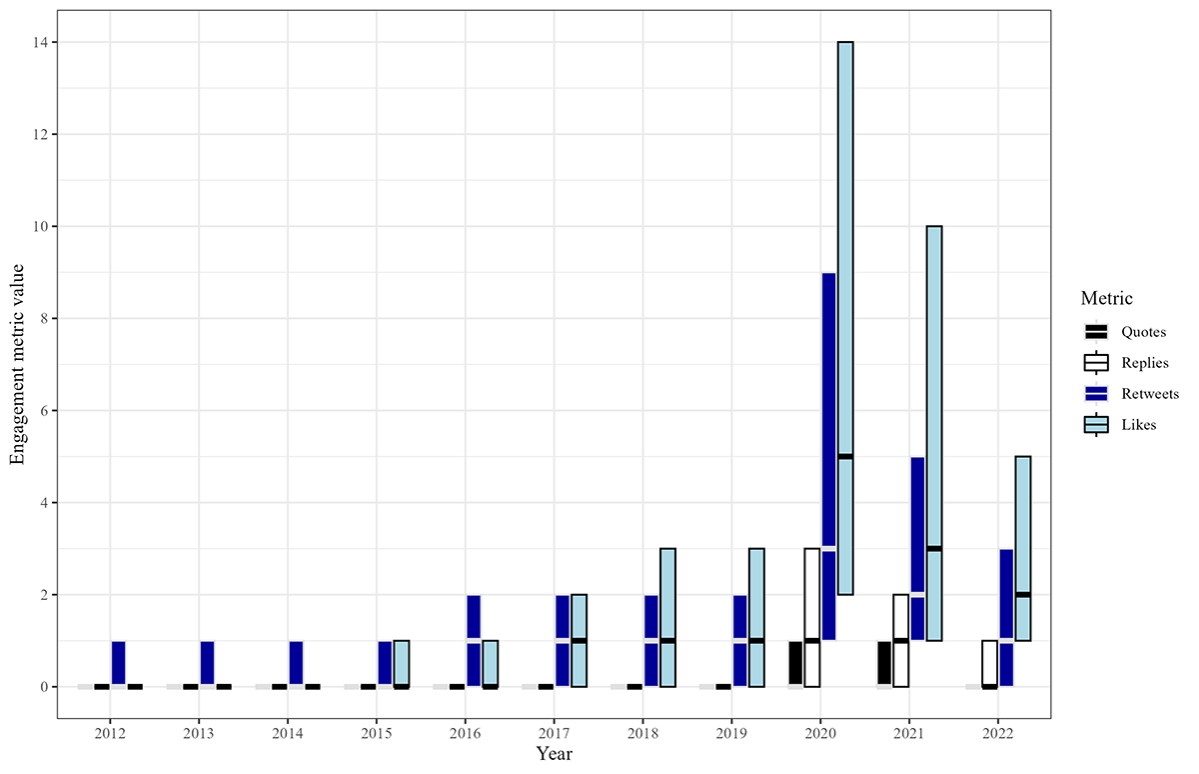
Among an exhaustive enumeration of non-retweets from US state public health agency accounts, grouped bar charts showing the IQR of audience engagement metrics, for each year from 2012 through 2022. The engagement metrics include quote tweets, replies, retweets, and likes. Note: medians are marked with a thick horizontal line. The bottom edge of each boxplot marks the 25th percentile. The top edge marks the 75th percentile.

The percentage of non-retweets with 0 on all audience engagement metrics was 62% (12,965/21,023) in 2012, decreasing to 20% (6465/32,668) in 2019. It reached a minimum of 6% (4123/66,507) in 2020 and 12% (6306/51,950) in 2022. Figure 3 presents each year’s IQR for audience engagement on state agencies’ non-retweets (ie, replies, quote tweets, and standalone tweets), summarized in the text below.

The mean number of likes was <0.6 before increasing to 1.2 in 2016 and then 2.7 in 2019. Its maximum was 17.6 in 2020. It decreased to 5.4 in 2022. The median was 0 before rising to 1 in 2017. Its maximum was 5 in 2020. It decreased to 2 in 2022. The maximum (n=69,798) was on a 2020 tweet about social distancing in Ohio. The second highest (n=12,022) was on a 2020 tweet about face coverings in California.

The mean number of replies was <0.3 before rising to a maximum of 3.5 in 2020. It decreased to 1.8 in 2022. The median was 0 every year except for 2020 and 2021, when it was 1. The maximum (n=2003) was on a 2022 tweet about the COVID-19 pandemic and breastfeeding or chestfeeding from Washington. The second highest (n=1954) was on a 2021 tweet about face coverings in Alabama.

The mean number of retweets was <1 until 2015, rising to 1.8 in 2018. Its maximum was 10.9, in 2020. It decreased to 2.9 in 2022. The median was 0 until rising to 1 in 2016. Its maximum was 3 in 2020. It decreased to 1 in 2022. The maximum (n=39,950) was on the previously mentioned Ohio tweet. The second highest (n=4844) was on a 2020 tweet about COVID-19 cases in Florida.

The mean number of quotes was <0.3 until rising to a maximum of 1.9 in 2020 and decreasing to 0.6 in 2022. The median was 0 for the entire study period. The maximum (n=7041) was on the previously mentioned Ohio tweet. The second highest (n=2552) was on a 2020 tweet about raw meat in Wisconsin.

### Persistence

The percentage of non-retweets with at least 1 mention of an account other than the authoring state public health agency decreased from a maximum of 21% (5399/25,267) in 2013 before decreasing to 17% (4437/26,750) in 2014. It then went up and down within that range before dropping off to 13% (9661/72,124) in 2021 and a minimum of 10% (5402/51,950) in 2022.

Among non-retweets, the top-mentioned account each year was @CDCgov. Before 2018, the top 10 accounts typically included at least 2 accounts representing a company or a non-CDC federal government account. In 2018, 9 of the top 10 represented state government agencies or officials, with the top 10 maintaining a similar balance for several years. In 2022, the top 10 included 2 additional CDC accounts and 2 other federal agencies.

The overall percentage of manual retweets decreased from 7% (1729/25,276) in 2012 to 1% (427/32,800) in 2014, then to <0.1% (5/50,024) in 2017 and 0% (0/91,099) in 2021.

### Replicability

The percentage of tweets that were formal retweets increased from 17% (4263/25,276) in 2012 to its maximum of 32% (15,955/50,024) in 2017. It decreased to 29% (27,698/94,205) in 2019 and increased slightly in 2020 before decreasing to 17% (10,847/62,797) in 2022.

The percentage of tweets that mentioned a CDC account was 4.2% (1056/25,276) in 2012, increasing to a maximum of 7% (3559/51,132) in 2018 and decreasing to a minimum of 3.5% (2218/62,797) in 2022. The percentage of tweets retweeting a CDC account increased from a minimum of 2.1% (521/25,276) in 2012 to a maximum of 5.4% (2756/51,132) in 2018. It decreased 2.2% (1363/62,797) in 2022.

The percentage of quote tweets increased from 2% (877/41,730) in 2015 to 4% (2129/50,024) in 2017 before decreasing each year to a minimum of 2% (1106/62,797) in 2022. Table 1 displays the retweet and quote tweet data by year.

**Table 1:** Among an exhaustive enumeration of all tweets from US state public health agency accounts, the portions that were either retweets or quote tweets, as well as the portion mentioning or retweeting a CDC^a^ account.

Year	Retweets (%)	Quote tweets (%)	Tweets with ≥1 mention of a CDC account (%)	Retweets of a CDC account (%)
2012	16.9	—^b^	4.2	2.1
2013	17.5	—	4.7	2.4
2014	18.4	—	4.8	2.9
2015	21.8	2.1	4.4	3.2
2016	26.0	3.3	6.0	4.1
2017	31.9	4.3	6.1	4.8
2018	31.8	3.7	7.0	5.4
2019	28.9	3.7	5.4	3.6
2020	29.4	2.5	4.8	3.6
2021	20.1	2.2	3.7	2.6
2022	17.3	1.8	3.5	2.2

^a^CDC: US Centers for Disease Control and Prevention.

^b^Not applicable.

### Searchability

The percentage of tweets with at least 1 hashtag increased from a minimum of 36% (9019/25,276) in 2012 to a maximum of 58% (24,017/41,730) in 2015. It decreased to 38% (23,565/62,797) in 2022.

The hashtags #health, #healthde, #ebola, #vibriovulnificus, #zika, #flu, and #covid19 were each the most common hashtags at 1 point during the study period. The #flu and #covid19 hashtags were each the most common for 3 consecutive years. The top 10 hashtags each year included at least 1 reference to natural disasters or disease outbreaks like #sandy or #ebola. From 2012 through 2016, top hashtags included at least 1 reference to a public health event, such as #cdcchat. In 9 years, the top 10 included #netde, a Delaware-specific hashtag.

Nine of the top 10 hashtags in 2020 and 2021 referred to the COVID-19 pandemic. This number decreased to 5 in 2022. In 2020, 5 of the top 10 hashtags were state-specific COVID-19 hashtags like #covid19ma. This decreased to 2 in 2021 and 2022.

**Figure 4:**
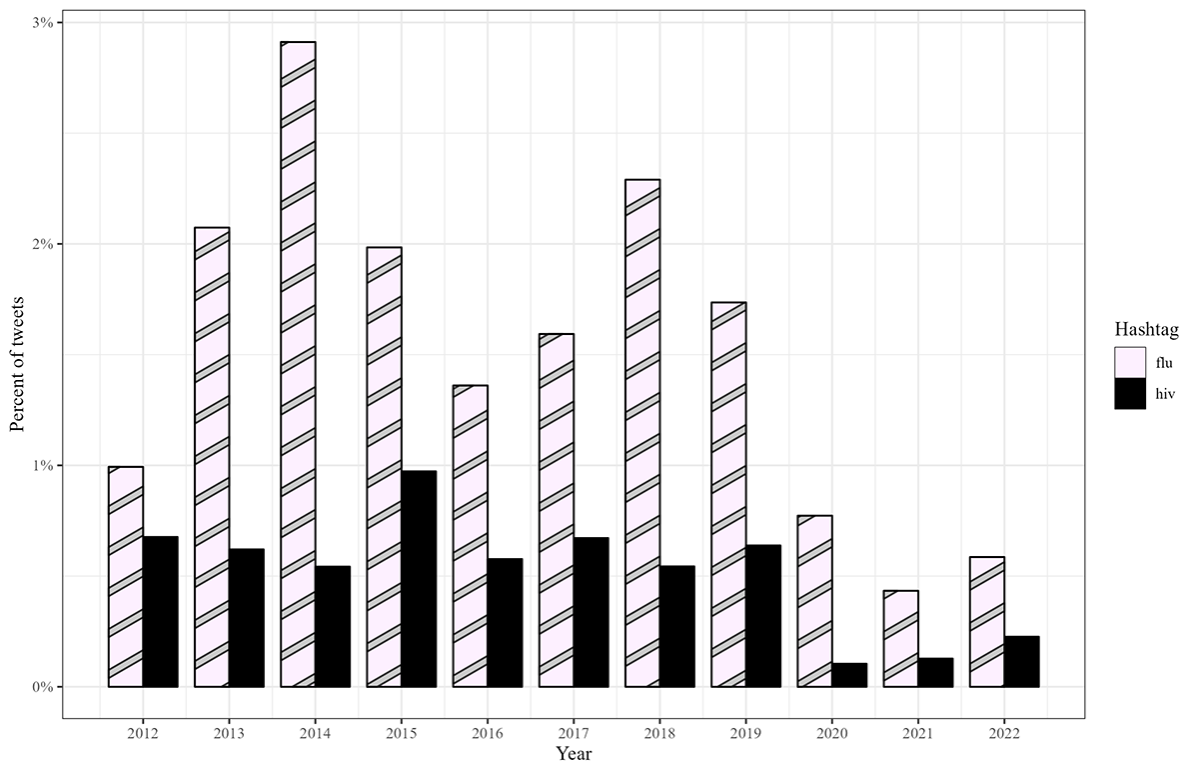
Among an exhaustive enumeration of tweets from US state public health agency accounts, grouped bar charts displaying the portion using the #flu or the #hiv hashtag, for each year from 2012 through 2022.

The hashtag #flu was in the top 10 every year in the study period. The hashtag #hiv was in the top 10 from 2012 through 2017 and in 2019. Figure 4 presents the percentage of tweets, including #flu or #hiv, each year.

## Discussion

### Principal Findings

We analyzed state public health agencies’ English-language tweets from 2012 through 2022 to provide a complete snapshot of Twitter use in the long term. We used Twitter API data as indicators for agencies’ engagement with key qualities of communication in a networked public: scalability, persistence, replicability, and searchability. We found increasingly fragmented communication over time, with less emphasis on replicability and persistence. This involved a drop in public interactions with other accounts in the form of retweets and quote tweets, which coincided with a dramatic increase in overall tweet volume and audience engagement during the COVID-19 pandemic.

Indicators of scalability and searchability on Twitter display a drastic shift at the beginning of the pandemic. We observed a dramatic increase in Twitter outputs and a focus on state-specific searchability amid a decreased focus on searchability overall. Though some states were especially active, the entire interquartile range of states’ mean Tweets per day shifted up throughout the pandemic. Audience engagement metrics followed a similar pattern, though still modest by marketing standards. Multiple COVID-19 hashtags were prominent from 2020 through 2022, with state-specific pandemic hashtags especially prominent in 2020. This contrasted with the prepandemic prominence of broader, more singular health emergency hashtags like #measles or #ebola.

Indicators of persistence and replicability did not display the same kind of COVID-19 pandemic–era shift. We observed more fragmented public health communication over time, relying less on replicating messages and traceable interactions with other accounts, on a time scale that did not align with the beginning of the pandemic. Mentions of state government officials became more prominent before the pandemic and remained so during the pandemic. The decrease in mentions of external accounts did not occur at the start of the pandemic. The rate of quote tweets decreased over time, starting before the pandemic. The rate of formal retweets followed a similar pattern but with a larger sudden decrease during the second year of the pandemic. The rate of mentions and retweets of CDC accounts decreased over time, starting before the pandemic.

Finally, an overarching finding in this study was between-state variation that did not fall neatly along the lines of geography, population, or a political binary of red and blue states. Florida, West Virginia, and Delaware were all highly active on Twitter for multiple years. Massachusetts, Wisconsin, and Ohio health agency communication styles included prominent use of state-specific pandemic hashtags. Tweets with record engagement were from Washington, Ohio, California, Florida, Wisconsin, and Alabama.

### Limitations

Our findings should be interpreted in light of 4 limitations. First, we collected our data retrospectively, meaning Tweet deletion and changes in privacy settings potentially shaped our findings. By the nature of these underlying nonrandom mechanisms, it is not possible to estimate the magnitude of potential missing data or how missingness may have changed over the study period. However, we argue this is not a critical issue for state health agencies, whose communications are part of the public record. This could, however, impact our findings around retweets and quote tweets of other accounts. We argue this is not likely a significant limitation, as the scale of “standalone tweets” we observed toward the beginning of our study period was similar to measures of “original tweets” from Thackeray et al [15]. A second limitation is our use of API indicators to filter tweets by language, relying on Twitter’s proprietary processing. Third, we focused solely on text features, meaning we missed trends in multimedia content, for example, reuploaded graphics. Finally, we studied only 1 platform. While our numerical results are not generalizable to other platforms, the patterns we identified in terms of our theoretical framework can be directly compared with data from other networked publics.

### Comparison With Previous Work

Our findings about the long-term prominence of self-replies and standalone tweets align with findings on the one-way nature of public health communication on Twitter from the previous decade [14,15]. We argue this could actually be a form of effective tailoring, for example, if known journalists draw information from Tweets for dissemination more often than they draw from press releases. However, there is a need for more research to make sense of our findings, such as exploring whether public health tweets during the pandemic have been helpful for these audiences.

Our findings differ from those of a study from Bradford et al [17], whose work covered a study period contained within our own. This appears to be due to their inclusion of only 1 Twitter account from Maine, in contrast with our inclusion of both Maine’s Department of Health and Human Services (DHHS) and its Center for Disease Control and Prevention (MeCDC) accounts. We made this decision after noting the MeCDC serves as the primary public-facing communication arm of the state DHHS. This aligns with the finding from Bradford et al [17] that a Maine account was barely active during their study period. Our conclusion about the overall volume of tweets also differs from Bradford et al [17], due purely to a matter of interpretation. Bradford et al [17] interpreted their observations as signs of low Twitter activity despite most accounts tweeting multiple times per day. We interpreted that same volume of tweets as high activity due in part to the sharp contrast with prepandemic levels. This highlights the need for more research on state health agencies’ social media strategies to help interpret our findings and those of other quantitative descriptions of public health communication on social media. Despite our differing conclusions, we echo the call of Bradford et al [17] for more research into the determinants of social media use at state health agencies. For example, we do not know to what degree our findings reflect purposeful decisions within state health agencies or the effects of changes in platform ownership, communication budgets, political factors, or platform design changes over time.

Our findings align with research on US state health departments' use of Facebook. Previous work has found the overall volume of Facebook posts by state health departments to vary [24]. Previous work has also identified different message frames between CDC and state and local health department Facebook posts [25], which could relate to our observed trends in decreasing engagement with CDC tweets and Twitter accounts in the latter half of our study period. Though Twitter and Facebook are prominent social media platforms, future research should quantitatively analyze state health departments’ use of other platforms as well.

### Conclusions

Our findings warrant further research to elucidate the factors driving the variation we saw between states and over time. This includes research into potential platform-specific communication strategies, health communication training, platform policy, and network norms. Such research should include qualitative content analyses of social media content to elucidate the actual use of social media in public health agencies, not just intended strategies. Further quantitative research should examine the impact of political factors on public health communication on social media. This includes state and federal political factors like budget changes and political divisions around the practice of public health. Further research is also warranted to better understand various audiences’ interactions with health agencies in networked publics, as well as their perceptions of state-level health communication.

Our findings raise further questions about what state health agencies’ communication should look like and how to support it. For example, it might be impractical for state agencies to target communities that have more contact with local health departments, even when there may be more extensive communication resources at the state level. There is not a robust national infrastructure to fill this gap, much less monitor and evaluate health communication on social media during a public health emergency. This gap is a critical contextual factor to consider when interpreting our findings around between-state variation.

Our study exemplifies the use of theoretical frameworks from media studies to understand trends in public health communication on social media. Though we examined metrics from one social media platform, our use of the networked public framework allows us to draw broader inferences about health communication. This increases the relevance of our findings despite differing features across time and across platforms. This is in contrast to the existing descriptive work of US state health agencies on Twitter. Integration of a theoretical framework should be a key part of future research on public health communication on social media, so as to extend its use beyond the limited scope of one platform’s features.

Finally, our study highlights the vulnerability of public health communication in networked publics. A social media platform’s use is subject to corporate decisions and community norms. If a platform shuts down or its communities deliberately hide health information, then public health investment in that platform may be suddenly irrelevant. For example, it is unclear whether the drop-off in tweets we observed in 2022 was the result of strategic decisions, insufficient resources, or platform changes. Even the ability to efficiently study public health communication largely depends on corporate policies around data access. Our study highlights the importance of supporting health communication research. This includes regulation to ensure academic access to social media data, as well as building a robust communication infrastructure outside of private platforms.

## Appendix

Multimedia Appendix 1 Strengthening the Reporting of Observational Studies in Epidemiology (STROBE) checklist.

Multimedia Appendix 2 Usernames of Twitter accounts included in this study.

Multimedia Appendix 3 Exhaustive summary data table.

## Abbreviations

API: application programming interface
CDC: US Centers for Disease Control and Prevention
DHHS: Department of Health and Human Services
MeCDC: Maine’s Center for Disease Control and Prevention
NIOSH: National Institute for Occupational Safety and Health
STROBE: Strengthening the Reporting of Observational Studies in Epidemiology

## Data Availability

The data analyzed during this study are available in a Harvard Dataverse repository [20]. The R code used to analyze the data and calculate descriptive statistics is available in an Open Science Framework repository [21].
